# Lidocaine can reduce the pain of intra-osseous fluid infusion

**DOI:** 10.1186/s13054-016-1359-5

**Published:** 2016-06-20

**Authors:** Jonathan Ilicki, Jesper Scholander

**Affiliations:** Department of Emergency Medicine, Karolinska University Hospital, 171 76 Solna, Sweden

We read the review by Petitpal et al on intra-osseous access (IOA) in adults with great interest [1]. The review was concise, and offers a comprehensive overview of IOA. However, we disagree with one key aspect.

The authors state that: “Despite a lack of evidence-based medicine, administration of lidocaine … has been proposed in conscious patients”. Two studies are cited to support that IOA is painful, and that it is unclear if lidocaine decreases pain associated with IOA. The first study is a case series (*n* = 26) which does not state if lidocaine was given and at what dose [2]. In the second study, 22 conscious patients received an intra-osseous (IO) needle. Of the 12 that received local anaesthesia, four were pain-free whereas the remainder experienced some pain. However, as the study did not report pain scores it is unclear but possible that the group with lidocaine experienced less pain [3].

A short-cut review from 2013 assessed the evidence for the use of local anaesthetics in IOA [4]. The review identified one conference abstract and one open-label study. Both were small (*n* = 10), open-label and manufacturer-sponsored, and compared different doses of lidocaine and insertion sites with regards to pain. Based on those findings the review concluded that injecting lidocaine both before and after flushing an IO needle is an effective method of reducing the pain of fluid infusion.

In clinical practice, using lidocaine for IO insertion in conscious patients takes little time and is unlikely to cause harm. We agree with the authors that there is a paucity of evidence, but the little research that does exist supports administering lidocaine in order to prevent pain.

## Abbreviations

IO, intra-osseous; IOA, intra-osseous access

## References

[1] Petitpas F, Guenezan J, Vendeuvre T, Scepi M, Oriot D, Mimoz O (2016). Use of intra-osseous access in adults: a systematic review. Crit Care.

[2] Cooper BR, Mahoney PF, Hodgetts TJ, Mellor A (2007). Intra-osseous access (EZ-IO) for resuscitation: UK military combat experience. J R Army Med Corps.

[3] Schalk R, Schweigkofler U, Lotz G, Zacharowski K, Latasch L, Byhahn C (2011). Efficacy of the EZ-IO needle driver for out-of-hospital intraosseous access—a preliminary, observational, multicenter study. Scand J Trauma Resusc Emerg Med..

[4] Stewart M (2013). Towards evidence-based emergency medicine: Best BETs from the Manchester Royal Infirmary. BET 1: local anaesthetics in intraosseous access. Emerg Med J.

